# The Use of Thermoporometry in the Study of Frost Resistance of Rocks

**DOI:** 10.3390/ma17030620

**Published:** 2024-01-27

**Authors:** Piotr Stępień, Edyta Spychał

**Affiliations:** Faculty of Civil Engineering and Architecture, Kielce University of Technology, Al. Tysiąclecia Państwa Polskiego 7, 25-314 Kielce, Poland; espychal@tu.kielce.pl

**Keywords:** rocks, frost resistance, DSC, thermoporometry, MIP

## Abstract

From an engineering point of view, it is important to know the factors influencing the frost resistance of rocks with porosity above 2% due to their different frost resistance. The article focused on frost durability research using the thermoporometry method (TMP) and the assessment of water phase transition in the pore spaces of selected rocks. For this purpose, the differential scanning calorimetry method (DSC) was used with the adoption of an original algorithm for eliminating the thermal inertia of the measurement system. The results of the DSC method were supplemented with the results of pore size distribution using the mercury intrusion porosimetry method (MIP) and standard rock frost resistance tests. Based on the research carried out using the thermoporometry method, it was confirmed that the ability of water to freeze in the temperature range from −5 °C to −20 °C was important, as well as the ability of rocks to increase the degree of water saturation during freeze–thaw cycles. Based on calorimetric tests combined with thermoporometry, in the case of non-frost-resistant rocks, a significant (dominant) share of pores with a radius of under 10 nm (amounting to over 0.008 cm^3^/cm^3^) was found. Pore connections in the water freezing process do not influence the investigated rocks’ frost resistance.

## 1. Introduction

Stone materials are one of the oldest raw materials used in construction. They have great potential as building stones, road stones, or raw materials for the production of building materials. Rocks are used as building cladding elements, pavement slabs, street curbs, substructures, concrete aggregate, etc. In historic buildings, these materials may be one of the main structural elements [1,2]. When this material comes into contact with water and is exposed to temperatures below 0 °C, information about its frost resistance becomes important. The frost resistance of rocks is primarily dependent on its pore space characteristics: pore volume, surface area, and pore size distribution [3]. The pore space characteristics determine the amount of water and the temperature at which phase transition takes place. One should bear in mind that rock materials are characterized by numerous varieties with individual physical and mechanical properties. They may differ significantly in the properties of their pore spaces, due to significant differences in the diagenesis process among not only one rock type but also rocks from one quarry [4,5,6]. For this reason, the possibility of using rocks from a given deposit should always be considered.

The water freezing process is controlled by two processes:

- The first of them is spontaneous nucleation. When the temperature is lowered below the phase transition temperature, liquid water does not immediately undergo a phase change. In supercooled water, small clusters of water molecules appear, forming the basis for crystal-initiating nuclei (homogenous nucleation) [7]. The spontaneous phase transition begins when the nuclei of the new phase reach a critical size “r^*^” [8,9]. In a water-filled pore, ice can only appear if the critical diameters of the nuclei are not larger than the pore diameter.

(1) $$r*=-\frac{2\gamma_{lc}}{\Delta G},$$

where: r* is the critical size of the nuclei; *γ_lc_* is the solid–liquid surface curvature; and Δ*G* is the thermodynamic potential.

The critical size of the nuclei decreases as the temperature decreases. As the temperature decreases below the phase transition temperature, the difference between the free energy of liquid water and ice (Δ*G*) based on bulk conditions (ignoring surface free energy) increases providing an ever-higher driving force for the transition. The probability of the appearance of ice in super cooled water is more likely the larger are the volume of free water and the lower the temperature compared to 0 °C. As Kozłowski [10] showed, the temperature of the spontaneous nucleation is a random phenomenon, unique even for the same system of physical and material factors.

- The second mechanism controlling the freezing process is derived by penetration of the ice front in pore spaces [11]. By removing energy from the system, an increasing volume of water gradually freezes. The freezing process of pore liquid after spontaneous nucleation is dependent on the liquid–ice interface and ice–gas interface [12,13,14]. When there is only liquid and solid water in the pores, the following equation can be used:
  (2) $$r_{K}=-\frac{2\cdot M\cdot \gamma_{lc}\cdot cos\theta }{\Delta H_{fus}\cdot \rho_{l}\cdot ln\left(\frac{T}{T_{0}}\right)},$$
  where: *r_K_* is the solid–liquid surface curvature; *M* is the molar mass of the liquid; *Θ* is the contact angle; Δ*H_fus_* is the heat of fusion; *ρ_l_* is the density of liquid; *T* is the temperature of phase transition; and *T_0_* is the phase transition temperature of the bulk liquid.

The freezing of water in pores is determined by pore interconnections and pore sizes [15,16]. This statement is in agreement with the research results of Morishige et al. [17], who studied the freezing and melting of water in mesoporous silica. They performed tests on two types of samples. In the first case, when pores of 10 nm and 17 nm were connected by interconnections with a diameter of approx. 4 nm, water freezing in pores larger than 4 nm occurred at approx. −41 °C. However, for the second case, when solidification and melting were studied in samples whose interconnections had diameters larger than 4 nm, exothermic effects occurred in a wider temperature range. For both cases, ice melting occurred in the temperature range from −13 °C to −7 °C and was independent of the radii of interconnections between pores and depended only on the internal radii of the pores [17].

A commonly used standard method for assessing the frost resistance of rocks is to subject them to freeze–thaw cycles and assess changes in their compressive strength, mass loss, and linear changes [18,19,20]. Important information on pore size distribution is also provided by the MIP method, supplemented by water absorption and capillary rise tests. In the case of the frost degradation process, methods based on weight loss, dynamic elastic modulus (by measuring the fundamental resonance frequency), compressive strength, visual inspection, and absorption measurements are primarily used. According to the standards [18,19,20] (slabs, setts, and curbs of natural stone for external paving), the absorption by weight for rocks should not exceed 3%, with a recommended absorption criterion of less than 0.5% [21,22]. The Polish standard [23] for external plinth slabs specifies the maximum permissible water absorption values by weight as 0.5% for granites, 1% for syenites, and 2% for sandstones. Standard guidelines [18,19,20,23] are based on limiting water absorption or specifying the maximum value of the decrease in mechanical properties in cyclic freeze–thaw tests; they do not take into account methods based on the phase change process. The recommended determination of frost resistance by performing 56 freeze–thaw cycles [18,19,20] may be not sufficient. As research by Martínez-Martínez et al. [24] shows, it may be necessary to perform more cycles than 56 to demonstrate differences in the frost resistance of rocks. Investigations in the case of rocks with above 10% open porosity values demonstrate that these rocks show a non-linear decay pattern, with long periods of apparent stability followed by rapid and catastrophic decay [24]. As Wessman [25] shows, for sandstones with porosity above 15%, destruction processes are observed already in the first freeze–thaw cycles. However, in the case of limestone and granite with porosity up to 4%, no damage was observed after 56 cycles [25]. As shown by the studies [26,27] for samples with porosity higher than 8.6%, there were significant changes in the mechanical properties and pore size distribution after the first few freeze–thaw cycles. Research by Nicholson et al. [28] shows that porosity under 2% can guarantee sufficient frost resistance. However, in the case of samples with porosities greater than 6%, there were those with good and poor frost resistance [28]. Research by Momeni et al. [29] shows that samples with porosity not exceeding 2% are characterized by a slight decrease in mechanical properties even after 300 freeze–thaw cycles. Benavente et al. [30] in their research on samples with porosity between 13 and 22% observed better frost resistance for rocks with greater water permeability, larger pore diameter, and smaller specific surface area.

Phase transition testing can be the basis for determining the frost resistance of rocks [4,31]. As Rusin et al. [32] show, rocks with capillary absorption above 1.5% are not frost resistant. In the case of rocks with absorption between 0.6 and 1.5%, the capability of water to freeze down to −10 °C takes a crucial role.

Additionally, the phase transition can be studied using dilatometric methods, and nuclear magnetic resonance (NMR) techniques [33,34,35].

An alternative to the above tests is the differential scanning calorimetry method and its use in thermoporometry research (TMP) [12,36,37]. Based on thermograms of heat released/supplied to the system, not only the ice content at specific temperatures can be calculated, but also the pore size distribution. The relationship between the temperature of phase transition and the pore radius in which the transition takes place is studied in several publications [12,17,36]. Differential scanning calorimetry was used in a variety of investigations for pastes or mortars [15,38,39,40], but it is not a typical method for testing rocks.

Despite general agreement on the high frost resistance of rocks with low porosity and water absorption, questions remain as to the factors influencing frost decay, especially in the porosity range between 2 and 12%. Therefore, in this paper the freezing and melting processes were examined for 7 rock specimens with porosity above 2% and three rocks with porosity below 2% (for comparison). The research in this article focused on using the thermoporometry method (TMP) and the designation and assessment of water phase transition in the pore space of selected rocks. Importantly, the calculations additionally took into account the inertia of the system. The algorithm described in the paper [41] was used to analyze the thermograms. This algorithm makes it possible to take into account the influence of the thermal inertia of the differential scanning calorimetry measurement system (DSC) on the recorded signals, for the part related to the melting of ice. The pore size distributions determined by the mercury intrusion porosimetry method (MIP) and the thermoporometry method (TMP) were compared. The pore size distributions obtained in this way from the MIP and TMP techniques were compared with the results of cyclic freeze–thaw tests, which enabled a more complete analysis and assessment of the frost resistance of the tested rocks. The article draws attention to the possibility of a broader analysis of phase changes in rocks based on the results of research using the DSC and TMP methods.

## 2. Materials and Methods

### 2.1. Materials

**Figure 1:**
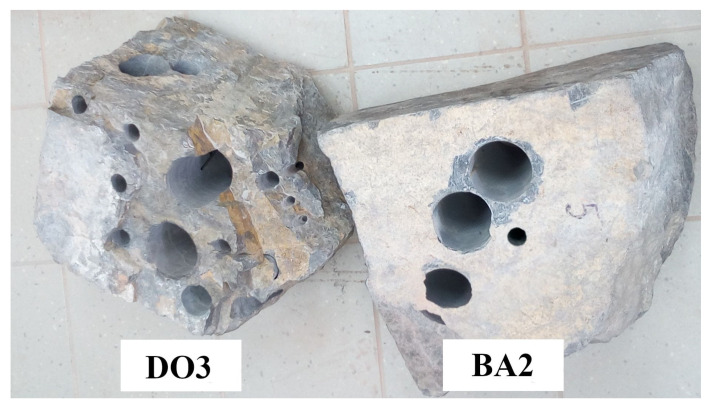
View of sample DO3 and BA2.

The research was performed on 10 rocks from different quarries. The designation and type of rocks are given in Table 1. The samples were drilled from one piece of rock, which eliminated the influence of differences in rock parameters from one deposit (see Figure 1).

Sample LIM1 comes from the Paleozoic core of the Świętokrzyskie Mountains (Poland), which formed in the Devonian period, while samples LIM2, LIM3, and LIM4 come from the southern and southwestern Mesozoic fringe of the Świętokrzyskie Mountains from the Jurassic period. The dolostones come from the Devonian period (Świętokrzyskie Mountains); DO1—the northwestern edge of the mountains, DO2 and DO4—the southwestern edge of the mountains, and DO3—the eastern edge of the mountains. The basalts (BA1 and BA2) come from the Fore Sudetic Block (Lower Silesia, Poland).

### 2.2. Differential Scanning Calorimetry (DSC)

Samples were core-drilled from rock specimens. The tests were conducted on cylindrical samples 13.5 mm in diameter and 70 mm in height. Before testing, the samples were dried at a temperature of 105 °C and saturated using the vacuum method with degassed distilled water. Thermal signals during cooling and heating were recorded using a BT 2.15 CS differential scanning calorimeter (SETARAM). The scanning program included cooling the sample from +20 °C to −80 °C, then after half an hour of stabilization at −80 °C, the sample was heated again to +20 °C. The scanning rate was 0.09 °C/min.

### 2.3. Mercury Intrusion Porosimetry (MIP)

After DSC tests, the samples were used for mercury intrusion porosimetry research (sample dimensions: 2.0 cm long, 1.4 cm diameter). Before testing, the samples were dried to a constant weight at 105 °C. The samples were then cooled to 20 °C in a desiccator. Pore size distribution, porosity, density, and bulk density were tested in an AutoPore IV model 9500 mercury porosimeter. Before testing, the samples were weighed and placed in the low-pressure port. The test included: (1) creating a vacuum in the penetrometer with the sample to the level of 2.6 Pa (20 μm Hg); (2) pouring mercury into the sample; (3) gradually increasing the pressure to 414 MPa while the apparatus measures the amount of mercury pressed; (4) gradually reducing the pressure to ambient values while the apparatus measures the amount of mercury exiting. For the calculations, the contact angle for mercury *ϴ* equal to 130° and the surface tension *γ_i_* equal to 0.485 N/m were assumed.

### 2.4. Frost Resistance Test

Rock samples for direct frost resistance tests with a diameter of 50 mm and a height of 160 mm were cut from one block of rock. Depending on the availability of rock samples, two or three samples were prepared for each rock. Samples were vacuum saturated with degassed distilled water. Specimens were subjected to 100 freeze–thaw cycles. A chamber with a programmable cycle was used for the tests. A single cycle included: cooling in air from +20 °C to –20 °C for 5 h, then heating in water at +20 °C for 3 h (the temperature sensor was located between the samples). After each block of 10 cycles, the sample mass and length change were measured, and visual damage was evaluated. Before weighing, the samples were removed from the water and wiped with a damp rubber. Length changes were determined by the Graf–Kaufman apparatus (each sample was equipped with measuring pins). The visual assessment of the samples was to determine any cracks, scratches, and damage.

## 3. Results and Discussion

### 3.1. Results of Differential Scanning Calorimetry Research and Thermoporometry Method

**Figure 2:**
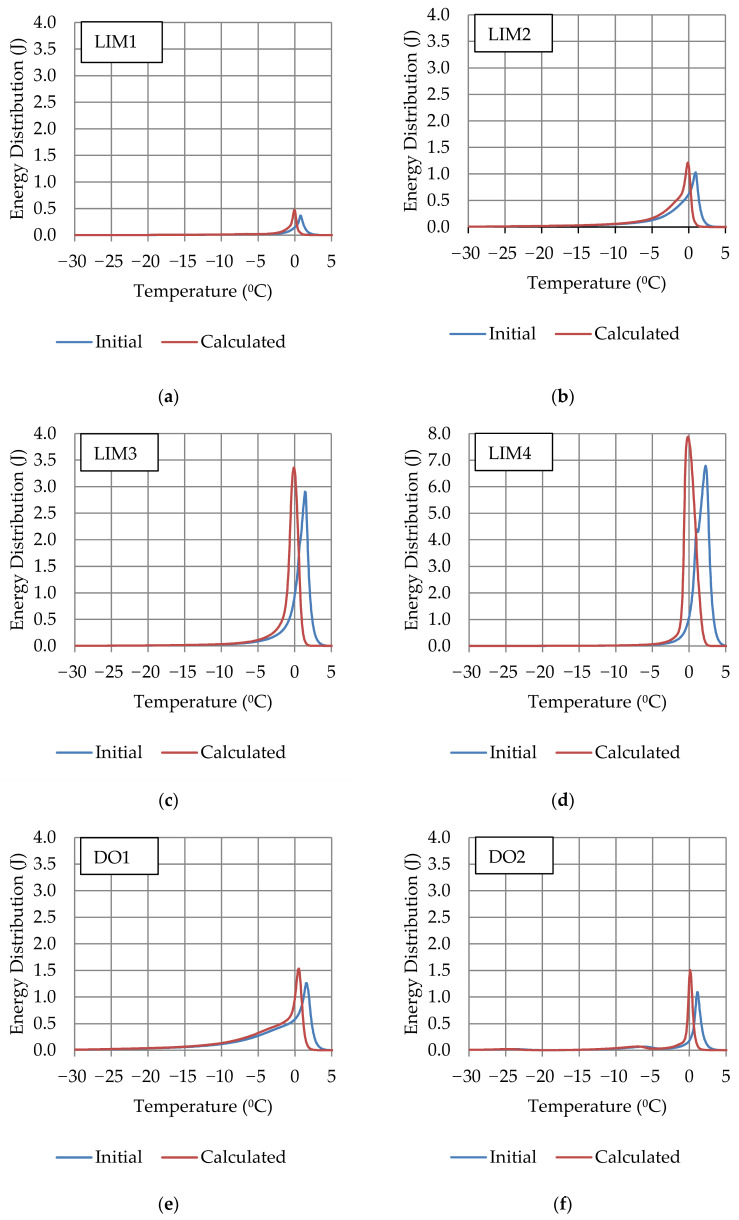

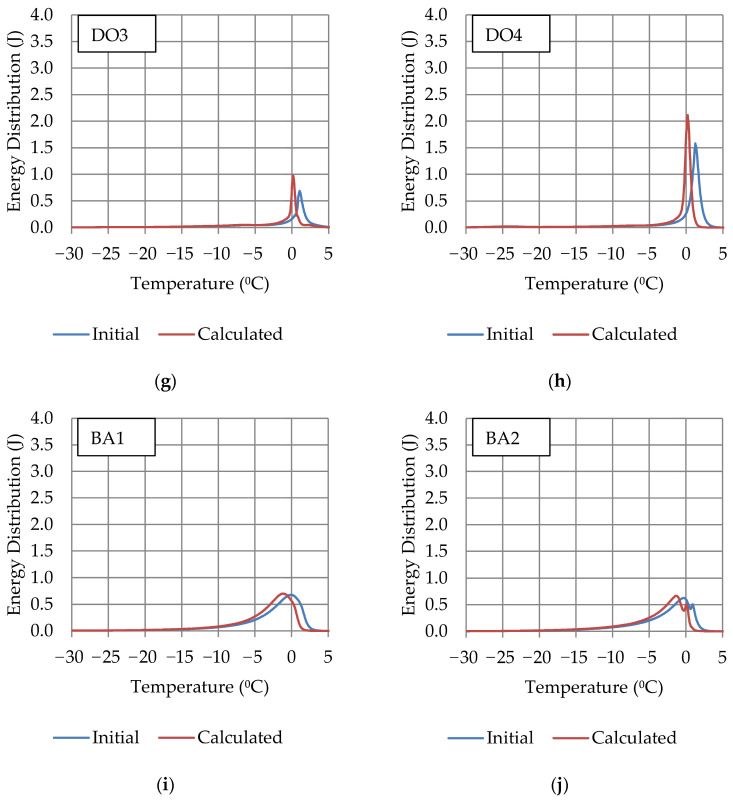
Energy distribution of samples: (**a**) LIM1; (**b**) LIM2; (**c**) LIM3; (**d**) LIM4; (**e**) DO1; (**f**) DO2; (**g**) DO3; (**h**) DO4; (**i**) BA1; and (**j**) BA2.

The phase change of water was examined using a differential scanning calorimeter. Of the obtained heat fluxes, the part related to the phase change of water and ice was separated. The recorded exothermic (during water freezing) and endothermic (related to ice melting) thermal effects were shifted due to the calorimetric measurement system’s inertia. For the endothermic part of the phase change energy distribution (related to the ice melting for the heating stage), the thermal inertia of the apparatus was taken into account using the algorithm described in the paper [41]. The exothermic part of the heat flows was left unchanged due to the initial stage of freezing. The energy distributions calculated by the above method are presented in Figure 2 (blue curve “initial”—energy distribution obtained directly from calorimetric measurements; red curve “calculated”—energy distribution obtained with algorithm taking account system thermal inertia).

Taking into account the inertia of the system (red line), we can better assign the phase transition energy to the temperatures at which it occurred. As a result, it is possible to estimate the pore size distribution in the tested material more precisely, which is a valuable justification and supplementation of knowledge about the frost resistance of rocks.

**Figure 3:**
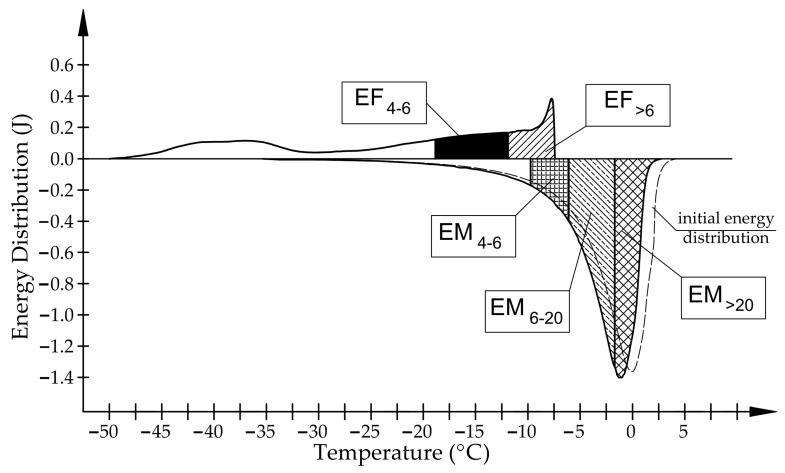
Energy distribution of phase transition for the BA1 sample.

For further analysis, the energy distributions for the endothermic and exothermic parts were divided as follows (see Figure 3):

*EF_>_*_6_ is the total energy corresponding to the freezing of water in pores with a radius above 6 nm, according to Equation (1).

*EF*_4–6_ is the total energy corresponding to the freezing of water in pores with a radius of 4 to 6 nm, according to Equation (1).

*EM*_4–6_ is the total energy corresponding to the melting of ice in pores with a radius of 4 to 6 nm, according to Equation (2).

*EM*_6–20_ is the total energy corresponding to the melting of ice in pores with a radius of 4 to 6 nm, according to Equation (2).

*EM_>_*_20_ is the total energy corresponding to the melting of ice in pores with radii above 20 nm, according to Equation (2).

For the relationship between the phase transition temperature and the radius of the pore (*r_p_*) in which this transition occurs, we applied the equation determined by Brun et al. [12].

(3) $$r_{p}=-\left(\frac{64.67}{\Delta T}\right)+0.57,$$

(4) $$r_{p}=-\left(\frac{32.33}{\Delta T}\right)+0.68,$$

where Δ*T* is the phase change temperature shift (Δ*T = T − T*_0_); *T* is the temperature of phase transition; and *T*_0_ is the phase transition temperature of bulk liquid (*T*_0_ = 0 °C).

**Figure 4:**
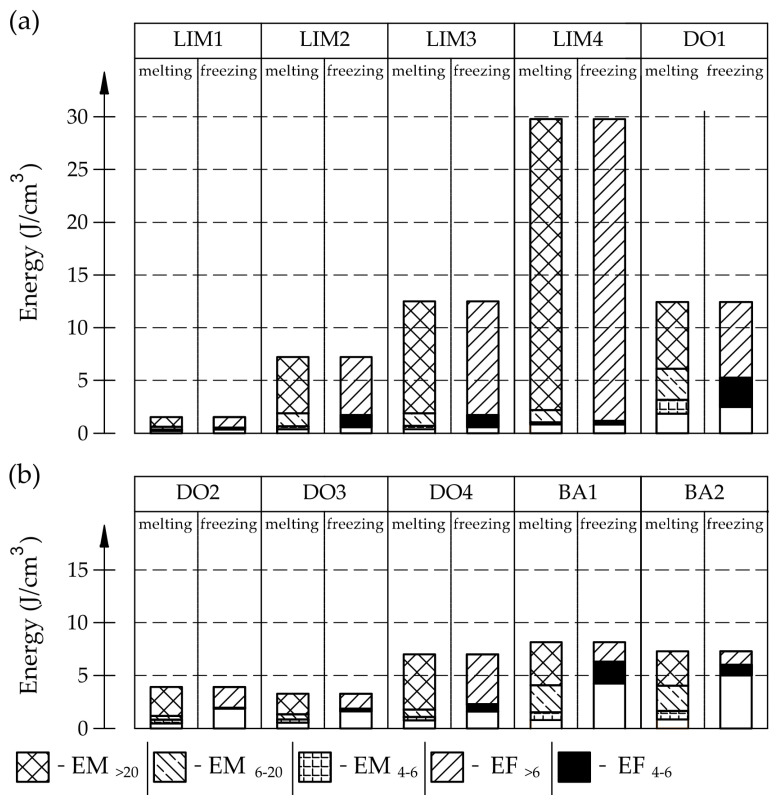
Energy values in particular intervals expressed per 1 cm^3^ of the sample mass: (**a**) dates for LIM1–LIM4 samples; (**b**) dates for DO1–DO4, BA1, and BA2 samples.

The energies divided in this way are shown in Figure 4.

In the case of basalts, the phase transition occurs in a much different way in comparison to the investigated carbonate rocks. There are significant shifts of the recorded signals related to water freezing to temperatures below −20 °C, potentially caused by the influence of interconnections on the freezing process.

### 3.2. Comparison Results of Differential Scanning Calorimetry Method and Thermoporometry with Results of Mercury Intrusion Porosimetry Method and Frost Resistance Test

Physical properties (bulk density, density, and porosity) of tested samples determined based on MIP are provided in Table 2. The bulk density of samples was in the range from 2.44 g/cm^3^ to 2.81 g/cm^3^, and density in the range from 2.70 g/cm^3^ to 2.94 g/cm^3^. The porosity of three rocks was below 2%; for others it was at least 2.59%.

Based on the tests conducted, it can be observed (see Figure 3 and Figure 4) that in the case of the examined igneous rocks (BA1, BA2), the way of freezing was significantly different from the way of water freezing in the pore space of carbonate rocks (LIM1–LIM4, DO1–DO4). More than 50% of water does not undergo the freezing phase change when the temperature decreases to −18 °C. Based on the pore size distribution from the MIP study and the endothermic part of the ice melt signals, at least 80% percent of the water should freeze (for the studied igneous rocks). In the case of limestone, the recorded amount of energy corresponding to the phase transitions of water at freezing in the pores bigger than 6 nm is similar to the amount of energy corresponding to the melting of ice (see Figure 3).

Based on the endothermic part of the energy distribution, the pore size distribution in which the phase transition occurred was determined. The accumulated pore volume obtained in this way, together with the accumulated pore volume obtained from the MIP test, are presented in Figure 5, Figure 6, Figure 7, Figure 8, Figure 9, Figure 10, Figure 11, Figure 12, Figure 13 and Figure 14.

The results of rock frost resistance tests, such as mass changes and length changes checked after subsequent freeze–thaw cycles, are presented in Figure 15, Figure 16, Figure 17, Figure 18, Figure 19, Figure 20, Figure 21, Figure 22, Figure 23 and Figure 24.

The LIM1, LIM4, DO2, and DO3 rocks (with a porosity of 1.34%, 9.70%, 1.99%, and 1.72%, respectively) were characterized by good frost resistance (based on the frost resistance test). In the case of the LIM2, LIM3, and BA2, rapid deterioration was observed after 90 freeze–thaw cycles. In the case of rocks BA1, DO1, and DO4, an increase in sample mass accompanied by gradual deterioration was observed. LIM4 was characterized by la ow increase of mass in relation to its porosity. The DO1 dolostone samples were damaged in the frost resistance test. This is potentially due to an additional significant amount of water freezing in the temperature range of −10 to −20 °C. The same applies to the studied basalts. For these samples, a slight increase in weight is recorded during subsequent freeze–thaw cycles. In the case of the LIM3 limestone, for which damage was recorded on the outer surface of the samples, there was a gradual increase in mass in cyclic freezing tests. In both the LIM3 limestone and the LIM2 and LIM4 limestones, the majority of the water freezes to a temperature of −20 °C. In the case of the LIM3 rock, a significant difference is observed in the cumulative pore volume obtained in the MIP and TMP tests. This may indicate that the pore space in this material is composed mostly of pores with radii larger than 40 nm, which are connected by constrictions with radii of approximately 10 nm.

**Figure 25:**
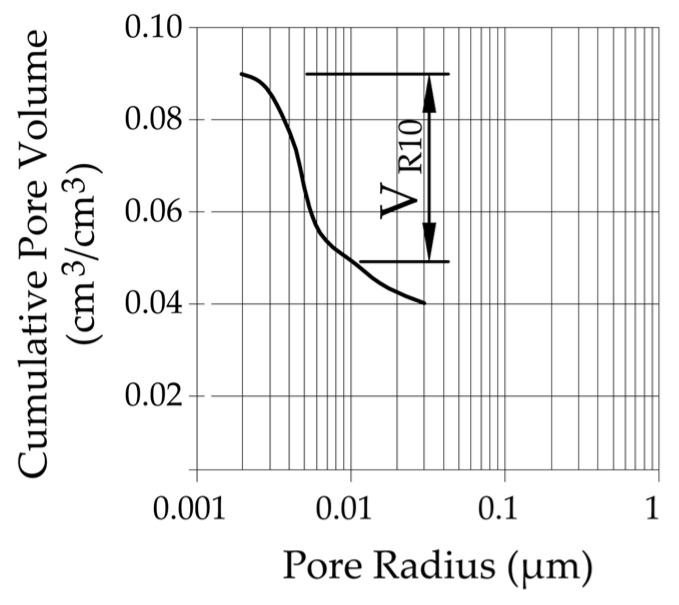
Graphical interpretation of the *V_R_*_10_ parameter.

To distinguish between frost-durable and not frost-durable rocks, we used the volume of pores with a radius below 10 nm calculated by the thermoporometry method (*V_R_*_10_ parameter). Figure 25 shows the graphical interpretation of the *V_R_*_10_ parameter.

**Figure 26:**
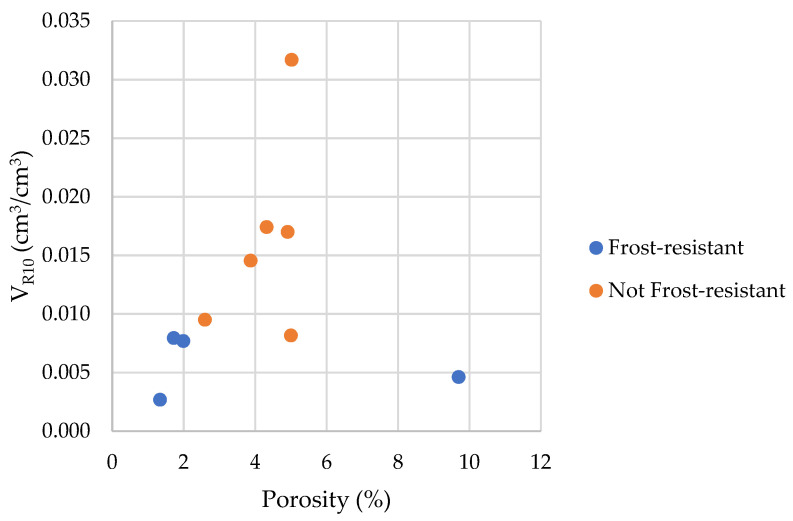
A comparison of the *V_R10_* parameter with the porosity of tested rocks.

In the case of frost-resistant rocks, the share of pores below 10 nm in which the phase transition of water occurred is less than 0.008 cm^3^/cm^3^ (see Figure 26). To determine the exact *V_R_*_10_ value that would distinguish rocks that do not show significant damage on the outer surface after 100 freezing cycles, tests should be conducted on a larger population, which will be the subject of future research.

Results of the cumulative pore volume *V_R_*_10_ and the information about the frost resistance of the tested rocks are introduced in Table 3.

**Figure 27:**
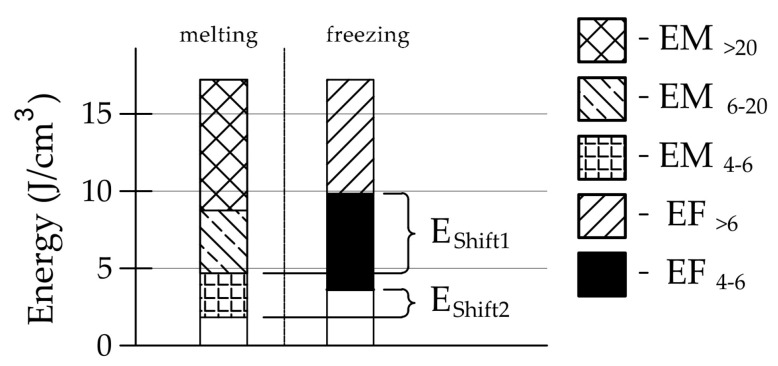
Graphical interpretation of the *E_Shift1_* and *Es_hift2_* parameters.

As described in the introduction, the occurrence of freezing effects can be attributed to two effects, pore connections or the presence of pore diameters corresponding to the freezing temperature according to Equation (2). To determine the influence of pore connections, it is possible to compare the process of water freezing and ice melting. For this purpose, we propose two factors *E_Shift_*_1_ and *E_Shift_*_2_ (Equations (5) and (6), see Figure 27).

(5) $$E_{Shift1}={EM}_{>20}+{EM}_{6-20}-{EF}_{>6},$$

(6) $$E_{Shift2}={EM}_{>20}+{EM}_{6-20}+{EM}_{4-6}-{EF}_{>6}-{EF}_{4-6},$$

where *E_Shift_*_1_ is the index of the influence of pore connections with a radius above 6 nm, and *E_Shift_*_2_ is the index of the influence of pore connections with a radius above 4 nm.

The bigger the values of *E_Shift_*_1_ and *E_Shift_*_2_, the more prominent the influence of pore connections on the freezing process. 

**Figure 28:**
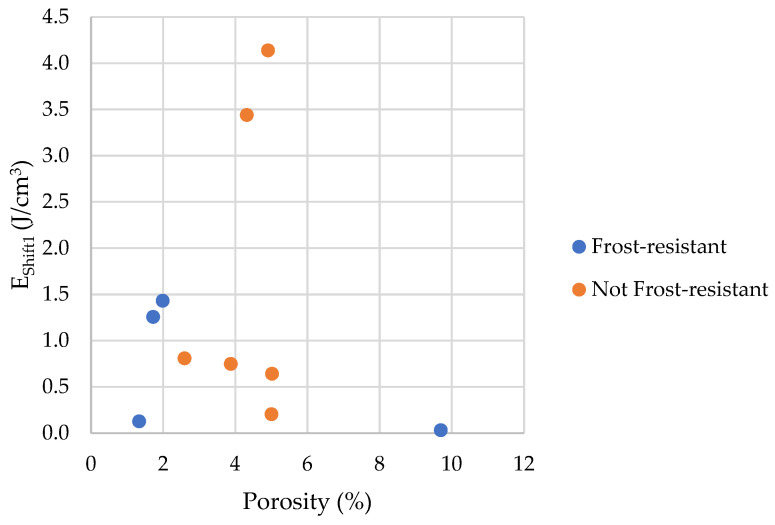
A comparison of the *E_Shift_*_1_ parameter with the porosity of tested rocks.

**Figure 29:**
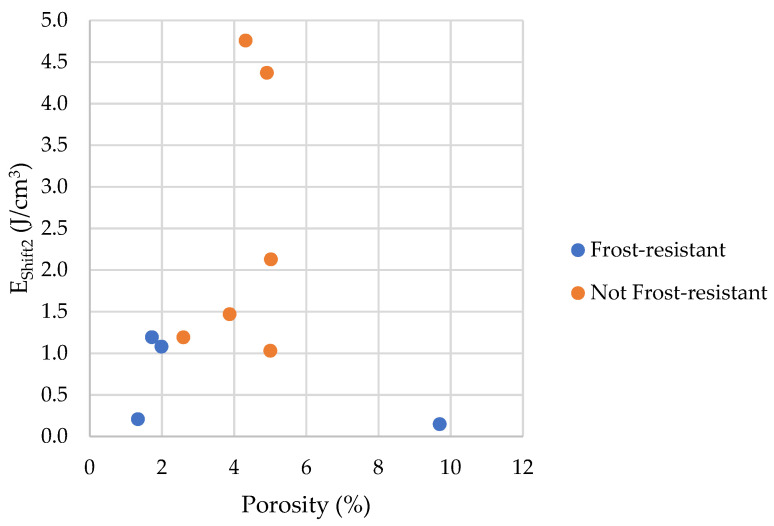
A comparison of the *E_Shift_*_2_ parameter with the porosity of tested rocks.

**Figure 30:**
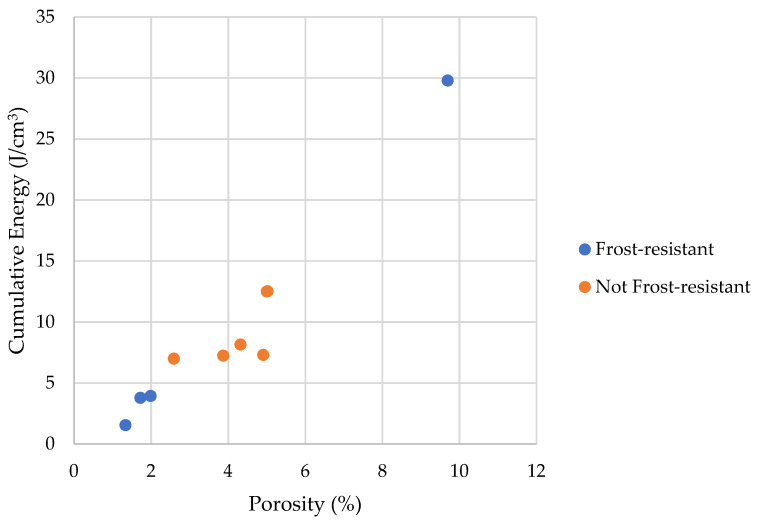
A comparison of cumulative energy with the porosity of tested rocks.

The Figure 28 and Figure 29 shows that the share of pores below 10 nm, in which phase transition can occur, has no significant impact on frost resistance. The frost resistance of a rock does not depend on the total amount of water that undergoes a phase change (Cumulative Energy—*E_SUM_*_,_ see Figure 30). The influence of interconnections between pores on the frost resistance of rocks remained an open question. Based on the values of the *E_Shift_*_1_ and *E_Shift_*_2_ indicators one can conclude that the influence of pore connection does not have a significant impact on their frost resistance.

No significant trend was observed between *V_R10_*, *E_Shift_*_1_, and *E_Shift_*_2_, the degree of pore filling, and porosity with the rate at which non-frost-resistant samples were damaged. From the above, it can be concluded that increasing the number of cycles to 100 as proposed in the literature seems reasonable to distinguish between frost-resistant and non-frost-resistant samples [21,24].

## 4. Conclusions

This paper focused on using the differential scanning calorimetry and the thermoporometry method for the assessment of water phase transition in the pore spaces of ten types of tested rocks. This analysis was supplemented with the results of pore size distribution using the mercury intrusion porosimetry method and the standard rock frost resistance research. The main conclusions of the study are as follows:

1. Frost resistance research of rocks was conducted on cylindrical samples subjected to 100 freeze–thaw cycles. In the case of samples LIM2, LIM3, BA1, BA2, DO1, and DO4, visible cracks and damage on the samples were observed during frost resistance tests. For the DO1 and BA1 samples, this is expected to be due to a significant amount of water freezing in the temperature range from –9 °C to –20 °C (Figure 4, *EF*_4–6_ value). The *EF*_4–6_ value for all non-frost durable samples was higher than 0.68 J/cm^3^.
2. The investigation confirms good frost resistance for rocks with porosity below 2% (LIM1, DO2, DO3). In the case of non-frost-resistant rocks, based on the analysis of test results from the differential scanning calorimetry and thermoporometry methods, a significant share of pores with a radius of less than 10 nm was found, with a larger value than 0.008 cm^3^/cm^3^. The LIM4 sample with a porosity of 9.70% has good frost resistance, which can be explained by it having a *V_R_*_10_ smaller than 0.008 cm^3^/cm^3^ and an insignificant amount of absorbed water during freeze–thaw cycles.
3. The use of algorithms taking into account the thermal inertia of the DSC measuring system may be a useful tool for assessing the pore space of rocks and interpreting the obtained results. The scanning calorimetry method (DSC) and thermoporometry method (TMP) are valuable supplements to information about the frost resistance of rocks determined by commonly used methods. The use of these methods allowed us to expand our knowledge about the frost resistance of rocks, especially those with porosity in the range of 2–12%. The usefulness of the DSC and TMP methods in assessing the phase transitions of the tested materials was indicated.

It is planned to focus on extending the research to a wider range of rocks.

## Figures and Tables

**Figure 5:**
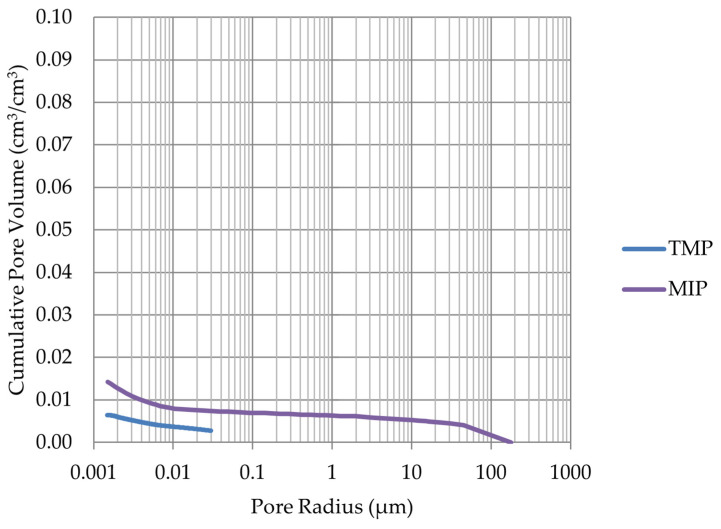
Cumulative pore volume—sample LIM1.

**Figure 6:**
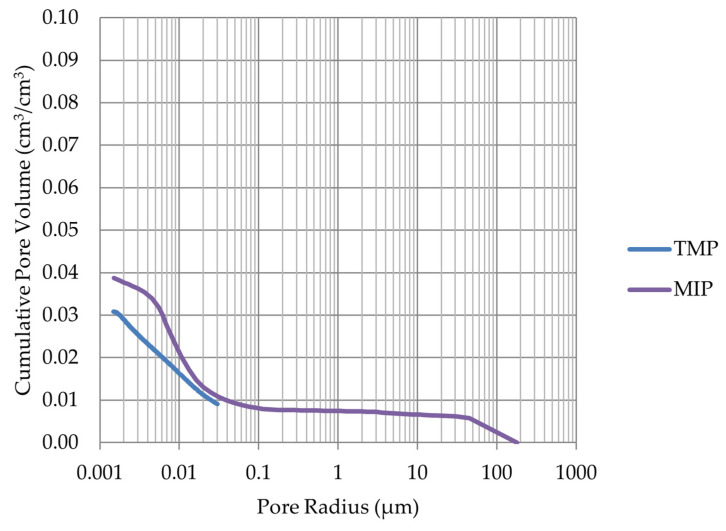
Cumulative pore volume—sample LIM2.

**Figure 7:**
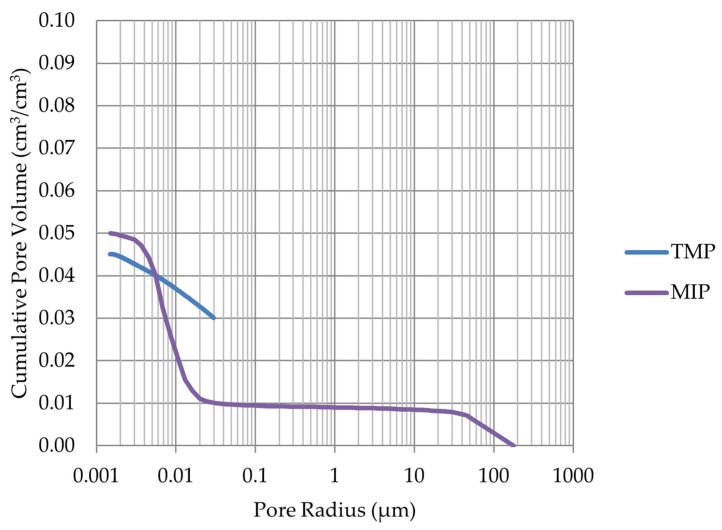
Cumulative pore volume—sample LIM3.

**Figure 8:**
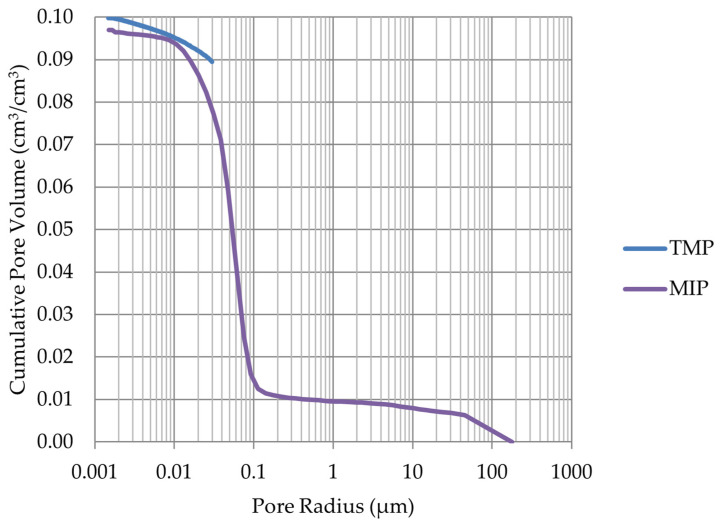
Cumulative pore volume—sample LIM4.

**Figure 9:**
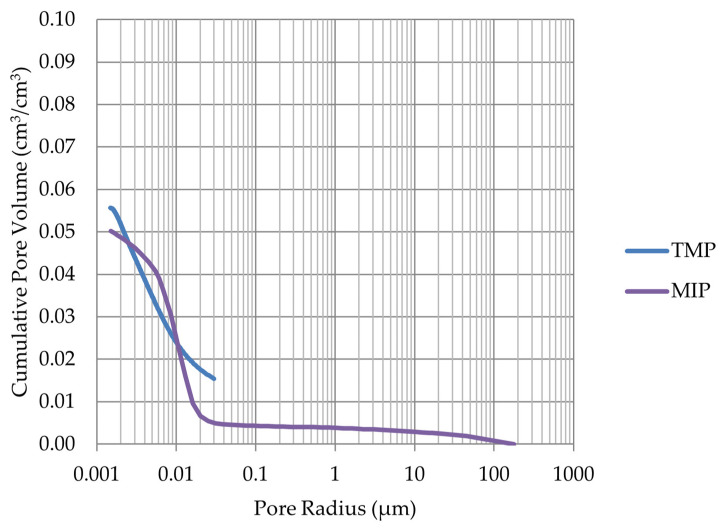
Cumulative pore volume—sample DO1.

**Figure 10:**
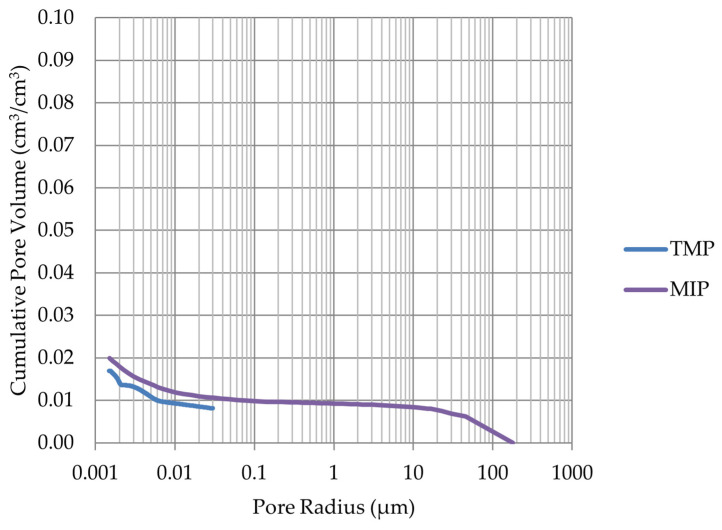
Cumulative pore volume—sample DO2.

**Figure 11:**
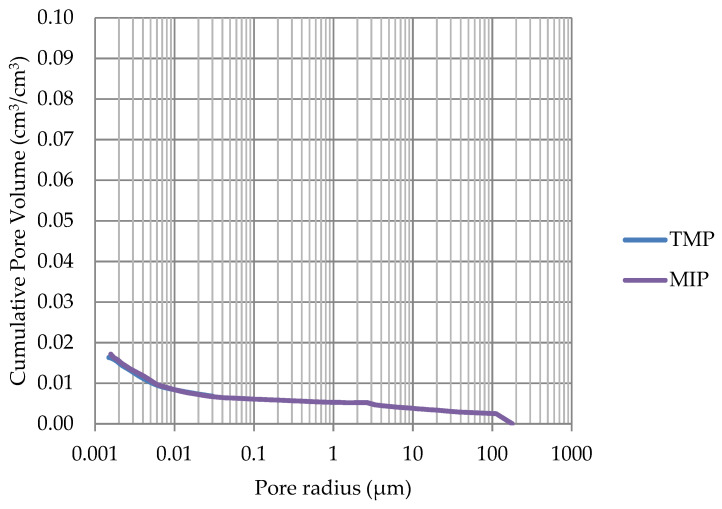
Cumulative pore volume—sample DO3.

**Figure 12:**
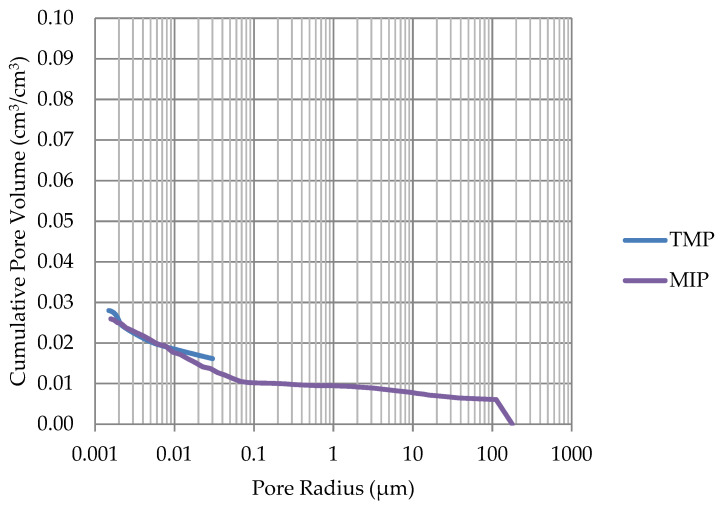
Cumulative pore volume—sample DO4.

**Figure 13:**
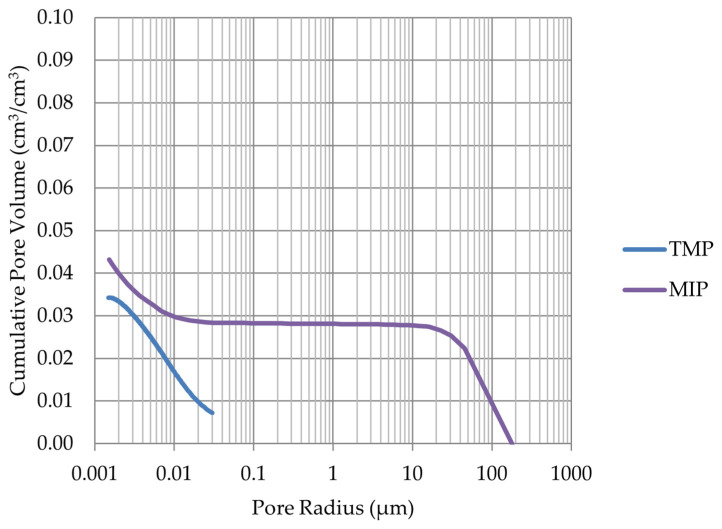
Cumulative pore volume—sample BA1.

**Figure 14:**
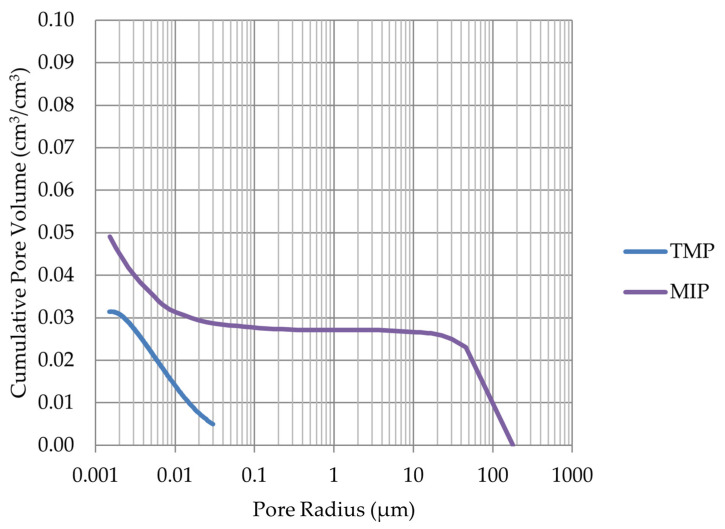
Cumulative pore volume—sample BA2.

**Figure 15:**
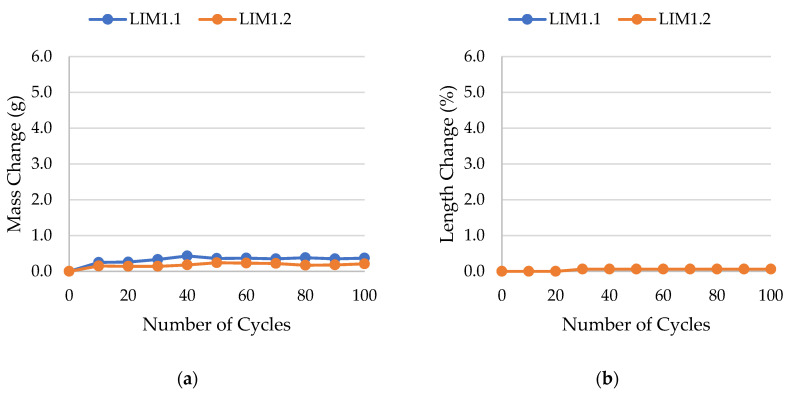
Result of the freeze–thaw test of sample LIM1: (**a**) mass change; (**b**) length change.

**Figure 16:**
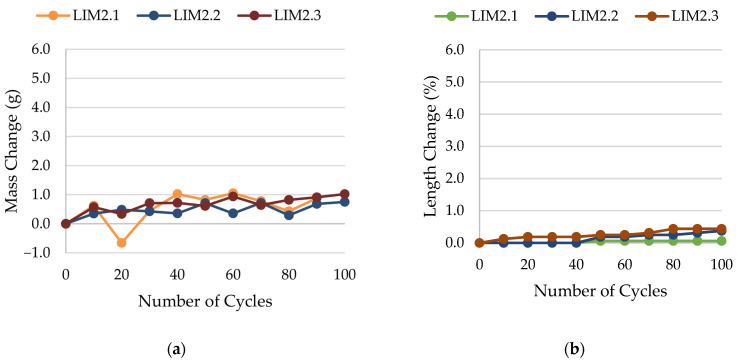
Result of the freeze–thaw test of sample LIM2: (**a**) mass change; (**b**) length change.

**Figure 17:**
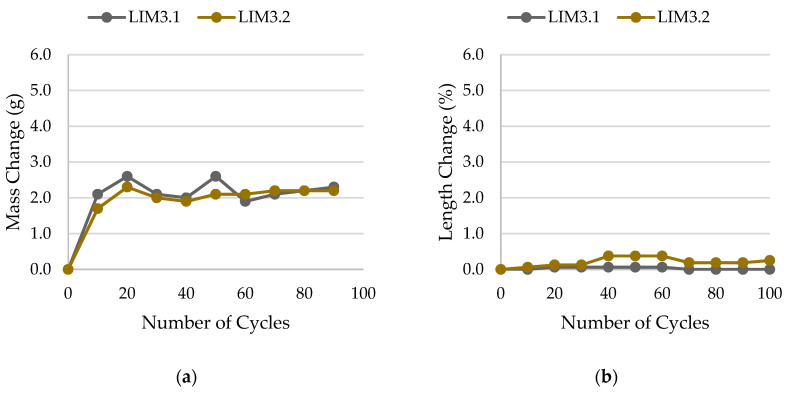
Result of the freeze–thaw test of sample LIM3: (**a**) mass change; (**b**) length change.

**Figure 18:**
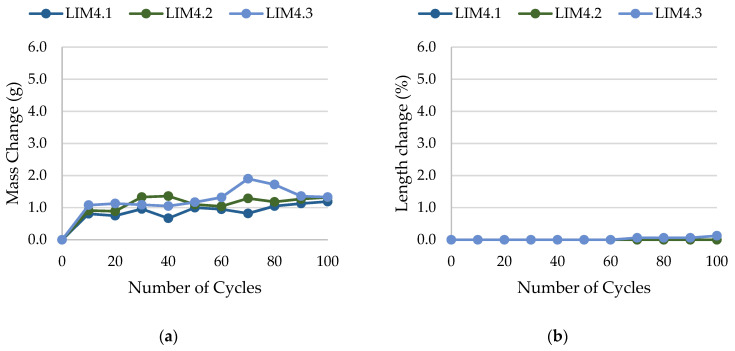
Result of the freeze–thaw test of sample LIM4: (**a**) mass change; (**b**) length change.

**Figure 19:**
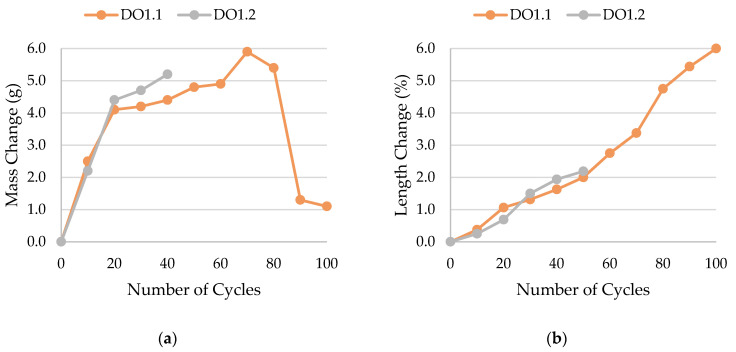
Result of the freeze–thaw test of sample DO1: (**a**) mass change; (**b**) length change.

**Figure 20:**
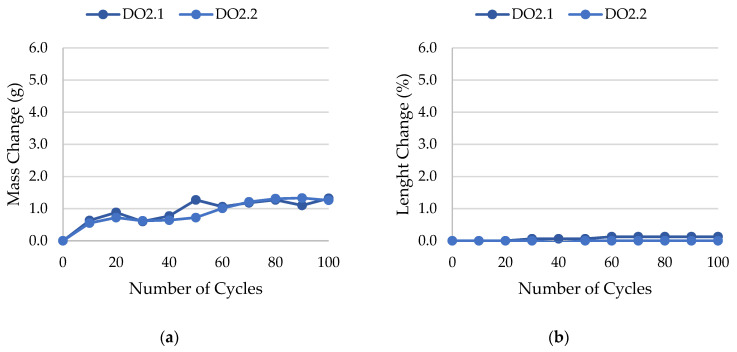
Result of the freeze–thaw test of sample DO2: (**a**) mass change; (**b**) length change.

**Figure 21:**
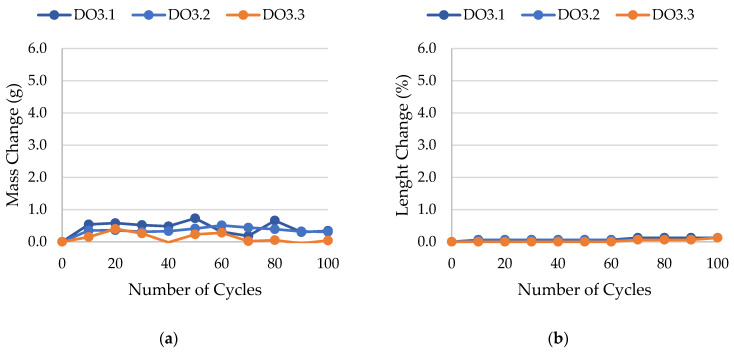
Result of the freeze–thaw test of sample DO3: (**a**) mass change; (**b**) length change.

**Figure 22:**
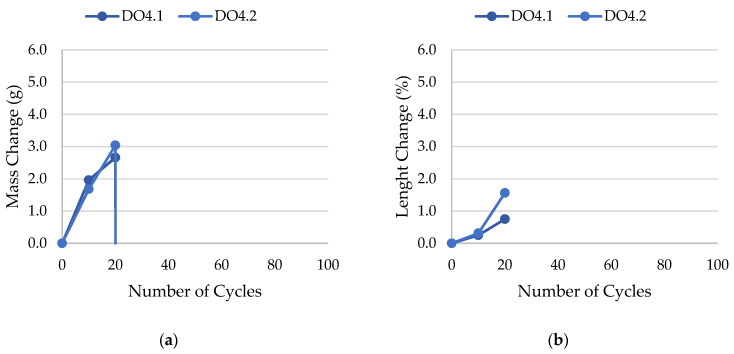
Result of the freeze–thaw test of sample DO4: (**a**) mass change; (**b**) length change.

**Figure 23:**
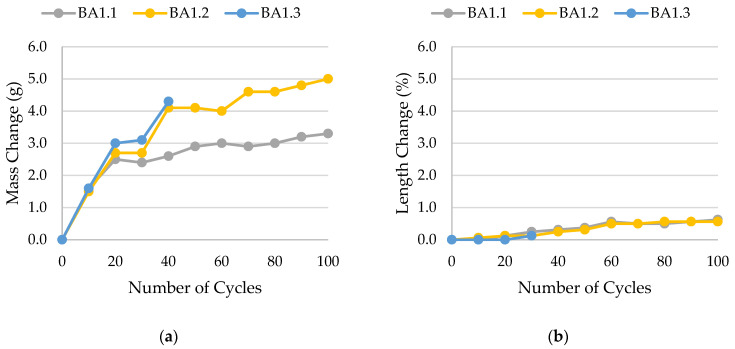
Result of the freeze–thaw test of sample BA1: (**a**) mass change; (**b**) length change.

**Figure 24:**
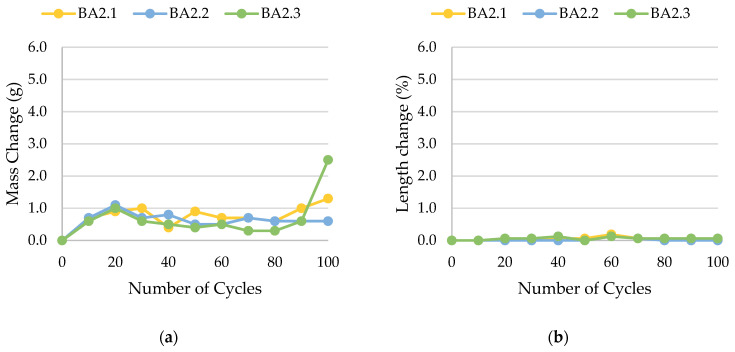
Result of the freeze–thaw test of sample BA2: (**a**) mass change; (**b**) length change.

**Table 1:** Types of rocks.

Rock Code	Rock Type	Dominant Minerals
LIM1	Devonian Limestone	100% Calcite
LIM2	Jurassic Limestone	100% Calcite
LIM3	Jurassic Limestone	100% Calcite
LIM4	Jurassic Limestone	90% Calcite, 10% Iron Compounds
DO1	Dolostone	90% Dolomite, 10% Calcite
DO2	Dolostone	60% Dolomite, 35% Calcite
DO3	Dolostone	87% Dolomite, 13% Calcite
DO4	Dolostone	60% Dolomite, 35% Calcite
BA1	Olivine Basalt	Plagioclase and Pyroxene, approx. 10% Hematite and Magnetite
BA2	Olivine Basalt	Plagioclase and Pyroxene, <10% Hematite and Magnetite

**Table 2:** Results of bulk density, density, and porosity tests of rocks based on MIP tests.

Rock Code	Bulk Density (g/cm^3^)	Density (g/cm^3^)	Porosity (%)
LIM1	2.68	2.71	1.34
LIM2	2.60	2.71	3.87
LIM3	2.57	2.71	5.00
LIM4	2.44	2.70	9.70
DO1	2.70	2.84	5.02
DO2	2.71	2.77	1.99
DO3	2.79	2.84	1.72
DO4	2.77	2.84	2.59
BA1	2.81	2.94	4.32
BA2	2.80	2.94	4.91

**Table 3:** Results of the cumulative pore volume and the determination of frost resistance of rocks.

Rock Code	Cumulative Pore Volume V_R10_ (cm^3^/cm^3^)	Information about Frost Resistance
LIM1	0.0027	frost-resistant
LIM2	0.0145	not frost-resistant, rapid decay after 90 freeze–thaw cycles
LIM3	0.0082	not frost-resistant, rapid decay after 90 freeze–thaw cycles
LIM4	0.0046	frost-resistant
DO1	0.0317	not frost-resistant, gradual decay
DO2	0.0077	frost-resistant
DO3	0.0080	frost-resistant
DO4	0.0095	not frost-resistant, gradual decay
BA1	0.0174	not frost-resistant, gradual decay
BA2	0.0174	not frost-resistant, rapid decay after 90 freeze–thaw cycles

## Data Availability

Data are contained within the article and supplementary materials.

## Abbreviations

DSC	differential scanning calorimetry method
*E_Shift_*_1_	the index of the influence of pore connections with a radius above 6 nm
*E_Shift_*_2_	the index of the influence of pore connections with a radius above 4 nm
*E_SUM_*	the total amount of water that undergoes a phase change in the DSC research
*EF_>_*_6_	the total energy corresponding to the contamination of water in pores with a radius above 6 nm
*EF*_4–6_	the total energy corresponding to the contamination of water in pores with a radius of 4 to 6 nm
*EM*_4–6_	the total energy corresponding to the melting of ice in pores with a radius of 4 to 6 nm
*EM*_6–20_	the total energy corresponding to the melting of ice in pores with a radius of 4 to 6 nm
*EM_>_*_20_	the total energy corresponding to the melting of ice in pores with radii above 20 nm
*M*	molar mass of the liquid
MIP	mercury intrusion porosimetry method
NMR	nuclear magnetic resonance techniques
*r**	critical size of the nuclei
*r_K_*	solid–liquid surface curvature
*r_p_*	pore radius
*T*	temperature of phase change
*T*_0_	freezing temperature of bulk liquid
TMP	thermoporometry method
*V_R_*_10_	cumulative pore volume of pores with radii under 10 nm
*γ* *_lc_*	solid–liquid surface curvature
Δ*G*	thermodynamic potential
Δ*H_fus_*	heat of fusion
*Θ*	contact angle
*ρ_l_*	density of liquid
